# Multiple administrations of bovine-appeasing substance during a 42-d preconditioning program followed by feedlot receiving and its effects on physiologic, health, and performance responses of feeder cattle

**DOI:** 10.1093/jas/skae151

**Published:** 2024-05-31

**Authors:** Keenan Kvamme, Rodrigo S Marques, Vinicius Alves Cruz, Arnaldo Limede Cintra, Makayla Anne Ogg, Sarah R McCoski, Christian J Posbergh, Amanda N Bradbery, Vitor R G Mercadante, Shea J Mackey, Autumn T Pickett, Reinaldo F Cooke

**Affiliations:** Department of Animal and Range Sciences, Montana State University, Bozeman, MT 59717, USA; School of Animal Sciences, Virginia Polytechnic Institute and State University, Blacksburg, VA, 24061, USA; School of Animal Sciences, Virginia Polytechnic Institute and State University, Blacksburg, VA, 24061, USA; School of Animal Sciences, Virginia Polytechnic Institute and State University, Blacksburg, VA, 24061, USA; Department of Animal and Range Sciences, Montana State University, Bozeman, MT 59717, USA; Department of Animal and Range Sciences, Montana State University, Bozeman, MT 59717, USA; Department of Animal and Range Sciences, Montana State University, Bozeman, MT 59717, USA; Department of Animal and Range Sciences, Montana State University, Bozeman, MT 59717, USA; School of Animal Sciences, Virginia Polytechnic Institute and State University, Blacksburg, VA, 24061, USA; Virginia-Maryland College of Veterinary Medicine, Large Animal Clinical Sciences, Blacksburg, VA 24061, USA; Department of Animal Science, Texas A&M University, College Station, TX 77845, USA; Department of Animal Science, Texas A&M University, College Station, TX 77845, USA; Department of Animal Science, Texas A&M University, College Station, TX 77845, USA

**Keywords:** appeasing substance, beef calves, performance, stress, weaning

## Abstract

This experiment evaluated the effects of multiple bovine-appeasing substance (BAS) administration during a 42-d preconditioning program followed by a feedlot receiving period on productivity, health, and physiological variables of feeder cattle. Ninety calves were weaned, weighed, loaded into a livestock trailer, transported for 70 km, and unloaded at the Bozeman Agricultural Research and Teaching Farm for a 42-d preconditioning program. Upon arrival, calf body weight (BW) was recorded again, and both pre- and post-transport BWs were averaged and used as calf weaning initial BW. Calves were ranked by BW, sex, and age in a completely randomized design and assigned to receive **1**) multiple administrations of BAS at weaning (day 0), days 14, 28, and before transport and feedlot entry (day 42; **BAS**; RSEA Group, Quartier Salignan, France; *n* = 9 pens/treatment), or 2) placebo (diethylene glycol monoethyl ether; **CON**; *n* = 9 pens/treatment). Treatments (5 mL) were applied to the nuchal skin area of each animal during the preconditioning period. Calves within treatment groups were ranked again by initial BW, sex, and age, in a manner that pens have similar initial BW, age, and three steers and two heifers and allocated to 1 of the18 drylot pens. On day 42, calves were combined within the treatment group, loaded into two different single double-deck commercial livestock trailers, and transported for 1,000 km (approximately 16 h). Upon arrival (day 43), calves were unloaded at the same feedyard. Blood samples were collected on days 0, 3, 7, 14, 21, 28, 42, 43, 46, 50, 57, 64, and 90. Average daily gain, final BW, and feed efficiency did not differ (*P* > 0.52) between BAS and CON calves in the preconditioning and receiving phases. A treatment × day interaction was detected (*P* < 0.001) for plasma haptoglobin concentrations, which was greater (*P* < 0.01) in CON on days 3 and 7 vs. BAS calves. During the preconditioning phase, serum NEFA concentration was reduced (*P* < 0.01) in BAS on day 3 compared with CON calves. A treatment × day interaction was detected (*P* = 0.001) for exit velocity, which was greater (*P* < 0.001) for CON vs. BAS calves on days 3, 7, 14, and 21 during the preconditioning phase and on day 46 of the receiving phase. Therefore, Applications of BAS reduced immunological responses and exit velocity associated with stress caused by management practices, but did not improve performance during the preconditioning and receiving phases.

## Teaser

Multiple administrations of a bovine-appeasing substance to recently weaned calves diminished an acute-phase response and exit velocity but did not improve performance during the preconditioning and receiving phase.

## Introduction

Weaning, road transport, and feedlot admission are the three most stressful events that feeder cattle encounter (Cooke, 2017). These inevitable management practices elicit inflammatory and acute-phase responses, often impairing cattle growth and health (Marques et al., 2012; Cooke, 2017). To mitigate the cumulative stress caused by these practices and to enhance cattle health during feedlot receiving, a preconditioning program is recommended from weaning to feedlot entry (Wieringa et al., 1974; Hilton, 2015). These initiatives provide the chance to employ management measures to prepare weaned beef calves for the stress and immunological challenges associated with long transit and feedlot entrance (Roeber et al., 2001; Duff and Galyean, 2006). Hence, preconditioning strategies to mitigate stress elicited by the regular management process are warranted to promote calf growth and welfare in cow-calf and feedlot systems.

One example includes the application of appeasing substance, initially discovered in swine and shown to affect the physiology and behavior of piglets (McGlone and Anderson, 2002; Archunan et al., 2014). Bovine-appeasing substance (BAS) is a mixture of fatty acids that imitate the composition of the original appeasing substance produced by cows (Pageat, 2001; Cooke et al., 2020). Accordingly, recent research reported that BAS administration to beef calves at weaning and beef bulls upon feedlot arrival improved initial body weight (BW) gain (Cappellozza et al., 2020; Colombo et al., 2020; Cooke et al., 2020; Fonseca et al., 2021). Cappellozza et al. (2020) also reported that BAS administration to beef calves at weaning improved average daily gain (ADG) and final BW during a 45-d preconditioning period. Along with the performance benefits, calves receiving BAS have a transient decrease in plasma cortisol response (Colombo et al., 2020), improved humoral immunity against bovine respiratory disease (BRD) pathogens (Schubach et al., 2020), and alleviated the resultant acute-phase response during a preconditioning period (Cappellozza et al., 2020; Cooke et al., 2020).

Nonetheless, most of the previous research was conducted with *Bos indicus*-influenced cattle. Little is known about BAS administration to *Bos taurus* cattle during a 42-d preconditioning program followed by a feedlot receiving period. Moreover, Cooke et al. (2020) reported that BAS is active for 15 d after administration when the benefits on calf performance and immune response were observed. Osella et al. (2018) administered BAS to dairy cows weekly upon turn out to pasture and reported productive benefits throughout their 28-d experimental period. Perhaps multiple BAS administrations might be beneficial during a 42-d preconditioning program followed by a feedlot receiving period to extend beef calves’ growth and immune response (Schubach et al., 2020). Therefore, research is warranted to evaluate the potential of the bovine-appeasing substance (BAS) in preconditioning programs to prepare weaned beef calves to face stress and immune challenges related to feedlot entry and to optimize their health and productivity. Based on this rationale, we hypothesized that multiple BAS administrations during 42-d preconditioning would mitigate the stress caused by weaning and feedlot entry, improving vaccine efficacy, health, and productivity of beef calves. Hence, the objective of this experiment was to evaluate the impacts of multiple BAS administrations during a 42-d preconditioning program followed by a feedlot receiving period on productivity, health, and physiological variables of feeder cattle.

## Materials and Methods

This experiment was conducted at Montana State University, Bozeman Agricultural Research and Teaching Farm (BART; 45°39ʹ45.9″N, −111°4ʹ28.1″W). Experimental procedures involving animals were reviewed and approved by Montana State University Agriculture Animal Care and Use Committee (protocol #2021-AA03).

### Animals, experimental design, and diets

Ninety recently weaned Angus × Hereford calves were obtained from Montana State University Red Bluff Research Ranch (Norris, MT; 45°34ʹ36.7″N 111°38ʹ53.7″W). All calves were weaned and weighed (prior to transport), and no vaccination protocol was administrated. Calves were loaded into a livestock trailer (Legend 50’ cattle liner; Barrett LLC, Purcell, OK), transported for 70 km, and unloaded at the BART Farm for a 42-d preconditioning program. Upon arrival, calf BW was recorded, and both pre- and post-transport BW were averaged and used as calf initial weaning BW (BW = 217 ± 2.5 kg and age = 160 ± 1.4 d). Pre- and post-transport BW were averaged to minimize the variability of gut fill (Goodchild, 1985), considering that water and food withdrawal was not an option for this trial because it could initiate an inflammatory reaction, impacting the results of this study (Marques et al., 2012, 2019). Calves were ranked by BW, sex (being 36 heifers and 54 steers), and age in a completely randomized design and assigned to receive 1) multiple administration of BAS at weaning (day 0) 14, 28, and before transport and feedlot entry (day 42; **BAS**; Secure Cattle; IRSEA Group, Quartier Salignan, France; *n* = 9 pens/treatment; 5 calves/pen), or 2) placebo (diethylene glycol monoethyl ether; **CON**; *n* = 9 pens/treatment; 5 calves/pen). Calves were immediately segregated by treatment into one of two groups and processed again for treatment administration, with CON calves processed first to avoid cross-contamination during treatment application (Schubach et al., 2020). The CON treatment used herein is also known as Transcutol (Sigma-Aldrich, St. Louis, MO), and it is used as an excipient for the BAS active ingredients. The BAS active ingredient is based on a proprietary mixture of fatty acids, including palmitic, oleic, and linoleic acids, added at 1% of the excipient and estimated to remain in treated animals for 15 d, according to the manufacturer (Schubach et al., 2020). Treatments (5 mL) were applied topically to the hair of the nuchal skin area of each animal every 14 d during the preconditioning period (Cooke et al., 2020). Following treatment application, calves within treatment groups were ranked again by initial BW, sex, and age in a manner that pens have similar initial BW, age, and three steers and two heifers and allocated to 1 of the 18 drylot pens (5 calves/pen; being 3 steers and 2 heifers; 5 × 9 m). Pens were arranged in three rows of eight pens/row with approximately 15 m between rows. Rows were randomly assigned to BAS (3 pens/row) and CON (3 pens/row) with two empty pens between pens of different treatments to preserve distance (10 m) and avoid cross-contamination among treatment groups.

During the preconditioning program (days 0 to 42), calves had free-choice access to a total mixed ration (TMR) balanced to meet the minimum nutritional requirements according to NASEM (2016), including grass-mixed hay, ground corn, dried distillers grain, and a mineral-vitamin supplement (Table 1), and water. On day 0, calves were vaccinated against *Clostridium* and *Mannheimia haemolytica* (One Shot Ultra 7; Zoetis Florham Park, NJ), *infectious bovine rhinotracheitis virus*, *bovine viral diarrhea complex, parainfluenza-3 virus, and bovine respiratory syncytial virus* (Bovi-Shield Gold 5; Zoetis) and were administered an anthelmintic (Dectomax; Zoetis). On day 21, calves were re-vaccinated against *Clostridium* (Ultrabac 8; Zoetis), *infectious bovine rhinotracheitis virus, bovine viral diarrhea complex, parainfluenza-3, and bovine respiratory syncytial virus* (Bovi-Shield Gold 5; Zoetis). On day 42, calves were combined within the treatment group, loaded into 2 different single double-deck commercial livestock trailers, and transported for 1,000 km (approximately 16 h). Transport length and duration were selected to elicit the stress challenges of a long haul (Marques et al., 2012). Upon arrival (day 43), calves were unloaded at the same feedyard (BART) with the same pen distribution used before transport but allocated to different drylot pens. From days 43 to 90, calves were fed ad libitum a TMR diet based on the same ingredients used during the preconditioning program, whereas transitioned using a multiple-step-up diet program (Table 1). Three transition diets were used for 7 d per diet to transition calves to the final diet within 21 d of initiating the step-up program (forage: concentrate ratio of 32:68 from days 43 to 49, 28:72 from days 50 to 56, 24:76 from days 57 to 63, and 20:80 from days 64 to 90).

**Table 1.** Composition and nutritional profile of the TMR offered for ad libitum consumption to calves during the experiment^1^

Item	Preconditioning phase	Receiving phase			
	Days 0 to 42	Days 43 to 49	Days 50 to 56	Days 57 to 63	Days 64 to 90
*Composition, dry matter basis*					
Grass-mixed hay, %	32	32	28	24	20
Ground corn, %	34	34	35	37	39
Dried distillers grains, %	29	29	32	34	36
Mineral mix ^2^, %	5	5	5	5	5
*Nutrition profile* ^3^ *, dry matter basis*					
Total digestible nutrients, %	77.2	77.2	78.6	79.9	81.3
Neutral detergent fiber, %	36.9	36.9	35.2	33.2	31.2
Crude protein, %	14.6	14.6	15.4	16.1	16.8
Net energy of maintenance, Mcal/kg	1.90	1.90	1.94	1.98	2.02
Net energy of gain, Mcal/kg	1.25	1.25	1.28	1.32	1.36

^1^During the preconditioning program (days 0 to 42), calves had free-choice access to a total mixed ration (TMR). On day 42, calves were combined within treatment group, loaded into two different single double-deck commercial livestock trailers, and transported for 1,000 km (approximately 16 h). Upon arrival (day 43), calves were unloaded at the same feedyard and with the same pen distribution used before transport but allocated to different drylot pens. From days 43 to 90, calves were fed ad libitum a TMR diet based on the same ingredients used during the preconditioning program, whereas transitioned using a multiple-step-up diet program.

^2^Containing 4.5% Ca, 1.04% P, 3.5% NaCl, 1.02% K, 0.32% Mg, 277 ppm Cu, 1,190 ppm Zn, 4 ppm Se, 459 ppm Mn, 88.18 IU/kg of vitamin A, 8.81 IU/kg of vitamin D3, and 220.4 IU/kg of vitamin E, and 550 mg/kg of monensin (CHS Inc., Sioux Falls, SD).

^3^Based on wet chemistry procedures by a commercial laboratory (Dairy One Forage Laboratory, Ithaca, NY). Calculations for net energy for maintenance and gain used the equations proposed by NASEM (2016).

### Sampling and laboratorial analyses

Samples of TMR ingredients were collected weekly, pooled across weeks, and analyzed for nutrient content (Dairy One Forage Laboratory, Ithaca, NY, USA). All samples were analyzed by wet chemistry procedures for concentrations of crude protein (method 984.13; AOAC, 2006), acid detergent fiber (method 973.18 modified for use in an Ankom 200 fiber analyzer, Ankom Technology Corp., Fairport, NY; AOAC, 2006), and neutral detergent fiber using α-amylase and sodium sulfite (Van Soest et al., 1991); modified for use in an Ankom 200 fiber analyzer, Ankom Technology Corp.). Calculations for net energy for maintenance and gain used the equations proposed by Nasem (2016). The nutritional profile of TMR is described in Table 1.

Calf full BW was collected on day 0 (weaning), days 41 and 42 (prior to transport) and 43 (upon arrival), and on days 90 and 91. These values were used to calculate ADG within each phase. During the preconditioning and receiving phase, feed intake was recorded daily by measuring offers and refusals from each pen. Samples of offered and refusals feed were dried for 96 h at 50 °C in forced-air ovens for dry matter calculation. Feed intake of each pen was divided by the number of calves within each pen and expressed as kilogram per calf/day. Feed efficiency was calculated using each pen’s total BW gain and total feed intake during the experiment. Exit velocity was recorded on days 0 (weaning), 3, 7, 14, 21, 28, 42 (prior transport), 43 (feedlot arrival), 46, 50, 57, 64, and 90 according to procedures described by Cooke (2014). The chute used for this experiment was a hydraulic Silencer Chute (Commercial Pro model; Moly Manufacturing, Lorraine, KS).

Blood samples were collected on days 0 (weaning), 3, 7, 14, 21, 28, 42 (prior transport), 43 (feedlot arrival), 46, 50, 57, 64, and 90 via jugular venipuncture into commercial blood collection tubes (Vacutainer, 10 mL; Becton Dickinson, Franklin Lakes, NJ) containing or not freeze-dried sodium heparin for plasma and serum collection, respectively. All blood samples were collected prior to daily feeding, placed immediately on ice, centrifuged (2,500 × *g* for 30 min; 4 °C) for either plasma or serum harvest, and stored at −80 °C on the same day of collection. Plasma samples were analyzed for cortisol (radioimmunoassay kit #07,221,106, MP Biomedicals, Santa Ana, CA) and haptoglobin concentrations (Cooke and Arthington, 2013). Serum samples, however, were analyzed for NEFA concentration using a colorimetric kit (HR Series NEFA—2; Wako Pure Chemical Industries Ltd. USA, Richmond, VA) with the modifications described by Pescara et al. (2010) (Weary et al., 2008). Plasma samples collected on days 0, 28, 57, and 90 were analyzed for IGF-1 concentrations (SG100; R&D Systems, Inc., Minneapolis, MN). Serum samples collected on days 0, 21, 42, and 64 were analyzed for antibodies against *bovine respiratory syncytial virus* (#P00651-2; IDEXX Switzerland AG, Liebefeld-Bern, Switzerland), parainfluenza-3 virus (#P0652-2; IDEXX), and bovine viral diarrhea virus types I and II (#99-44,000; IDEXX).

The intra- and inter-assay CV were, respectively, 2.9% and 2.8% for haptoglobin, 2.2% and 3.3% for cortisol, 4.45 and 6.0% for NEFA, 2.0% and 3.9% for IGF-1, 4.1% and 7.8% for bovine respiratory syncytial virus, 2.9% and 7.5% for parainfluenza-3 virus, and 6.8% and 7.9% for bovine viral diarrhea viruses.

### Statistical analysis

All performance and physiological results were analyzed using the pen as the experimental unit (*n* = 9/treatment), the MIXED procedure of SAS (SAS Inst. Inc., Cary, NC), and the Satterthwaite approximation to determine the denominator degrees of freedom for tests of fixed effects using 16 °C of freedom. These data were analyzed using pen(treatment) and calf(pen) as random variables, but for intake and feed efficiency (G:F) were used pen(treatment) as the random variable as described by Colombo et al. (2020) and Schubach et al. (2020). Model statements for BW parameters and feed efficiency contained the effects of treatment. Model statements for feed intake, exit velocity, and blood variables contained the fixed effects of treatment, day, and all resultant interactions. Blood and exit velocity variables were analyzed using results from day 0 as an independent covariate. The specified term for all repeated statements was day, with pen(treatment) as the subject for intake and efficiency and calf(pen) as a subject for all other analyses. The covariance structure used was first-order autoregressive, which provided the smallest Akaike information criterion and, hence, the best fit for all variables analyzed. Results were reported as least square means or covariate-adjusted least square means for blood and exit velocity variables. Significance was set at *P* ≤ 0.05, and tendencies were determined if *P* > 0.05 and ≤ 0.10.

## Results

**Figure 1.**
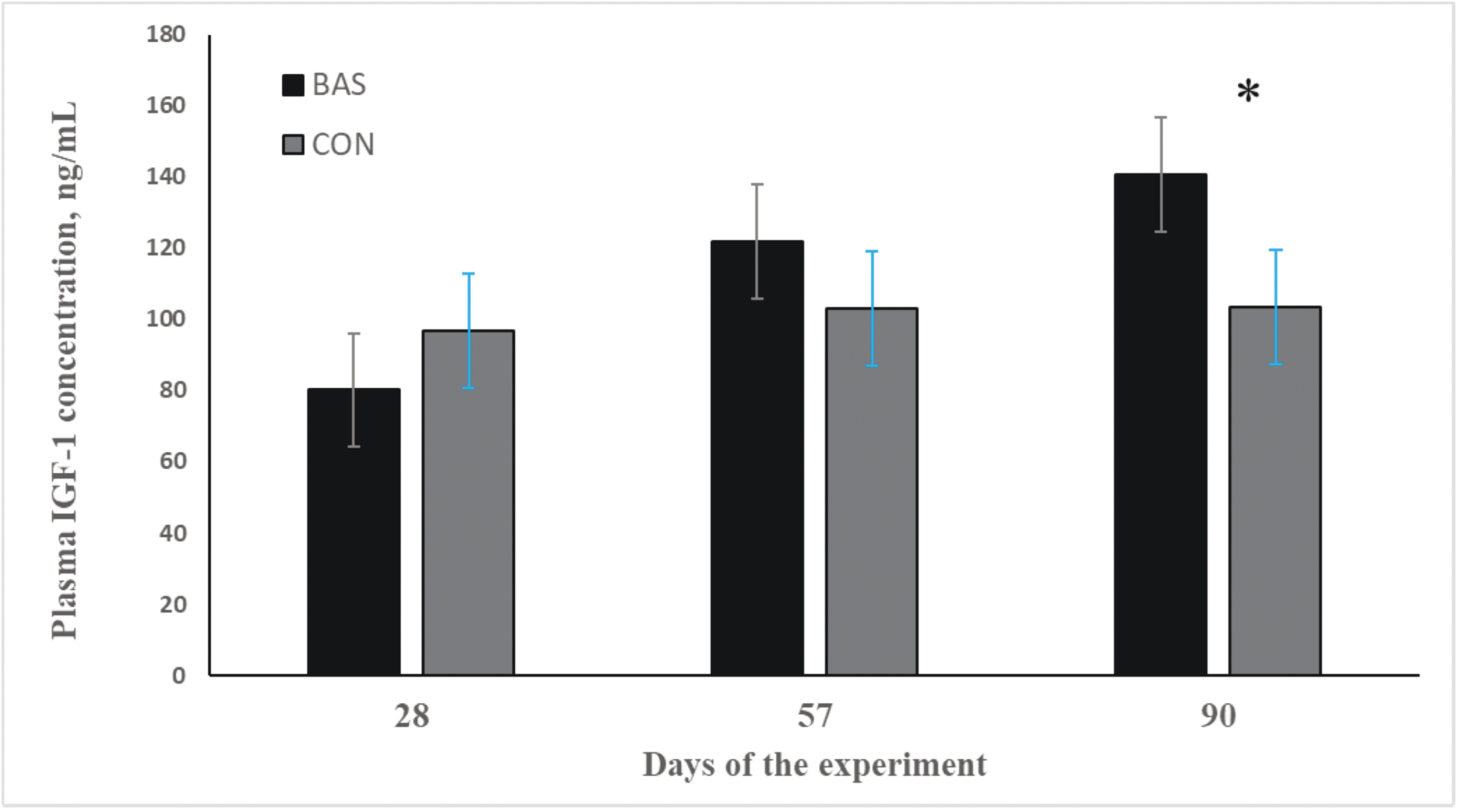
Plasma IGF-1 concentration of beef calves administrated multiple bovine-appeasing substances (**BAS,***n* = 9) or not (**CON**, *n* = 9) every 14 d during a 42-d preconditioning followed by a feedlot receiving phase (days 43 to 90). Calves individually received 5 mL of a BAS (IRSEA Group, Quartier Salignan, France) or CON (diethylene glycol monoethyl ether) topically to their nuchal skin area on days 0, 14, 28, and 42. Values obtained from day 0 were used as an independent covariate within each experimental period. Therefore, the results reported are covariately adjusted least square means using the pen as the experimental unit (*n* = 9/treatment), and the treatment effect was tested with 16 °C of freedom. A treatment × day interaction was detected (*P* = 0.04). Within day, *P < 0.01.

As designed, calf initial BW was similar (*P* = 0.99) between treatments. ADG and final BW did not differ (*P* > 0.52) between BAS and CON calves in both preconditioning and receiving phases (Table 2). No treatment effects were also detected (*P* > 0.44) for daily TMR intake in any of the phases (Table 2). Overall feed efficiency also did not differ (*P* > 0.54) between BAS and CON calves in both the preconditioning and receiving phases (Table 2). Nonetheless, a treatment × day interaction was detected (*P* < 0.001) for IGF-1 concentration, which was greater (*P* < 0.01) in BAS on day 90 compared with CON calves (Figure 1).

**Table 2.** Performance parameters of beef calves administered multiple bovine appeasing (**BAS,***n* = 9) or not (**CON**, *n* = 9) every 14 d during a 42-d preconditioning phase followed by a feedlot receiving phase (days 43 to 90)^1^

Item	CON	BAS	SEM	*P-value*
*Preconditioning phase* ^2^				
Calves initial age, d	161	160	1.4	0.78
Initial body weight, kg	217	217	2.5	0.99
Final body weight, kg	271.4	271.0	3.5	0.93
Average daily gain, kg/d	1.28	1.27	0.07	0.92
Dry matter intake, kg/d	6.38	6.19	0.17	0.44
Feed efficiency ^3^	0.201	0.205	0.005	0.68
*Receiving phase* ^2^				
Initial body weight, kg	256.1	256.5	3.6	0.93
Final body weight, kg	347.8	350.6	3.5	0.58
Average daily gain, kg/d	1.91	1.96	0.05	0.53
Dry matter intake, kg/d	9.94	9.93	0.17	0.96
Feed efficiency ^3^	0.192	0.197	0.006	0.54

^1^Calves individually received 5 mL of a BAS (IRSEA Group, Quartier Salignan, France) or CON (diethylene glycol monoethyl ether) topically to their nuchal skin area on days 0, 14, 28, and 42. During the preconditioning program (days 0 to 42), calves had free-choice access to a total mixed ration (TMR). On day 42, calves were combined within treatment group, loaded into 2 different single double-deck commercial livestock trailers, and transported for 1,000 km (approximately 16 h). Upon arrival (day 43), calves were unloaded at the same feedyard and with the same pen distribution used before transport but allocated to different drylot pens. From days 43 to 90, calves were fed ad libitum a TMR diet based on the same ingredients used during the preconditioning program, whereas transitioned using a multiple-step-up diet program.

^2^Calf full BW was collected on days 0 (pre- and post- transport at weaning), 41 and 42 (prior to transport) and 43 (upon arrival), and on 90 and 91. These values were used to calculate average daily gain within each phase. Pen was the experimental unit (*n* = 9/treatment). Therefore, the treatment effect was tested with 16 °C of freedom.

^3^Feed efficiency was calculated using total BW gain, and total feed intake of each pen during each experimental period.

**Figure 2.**
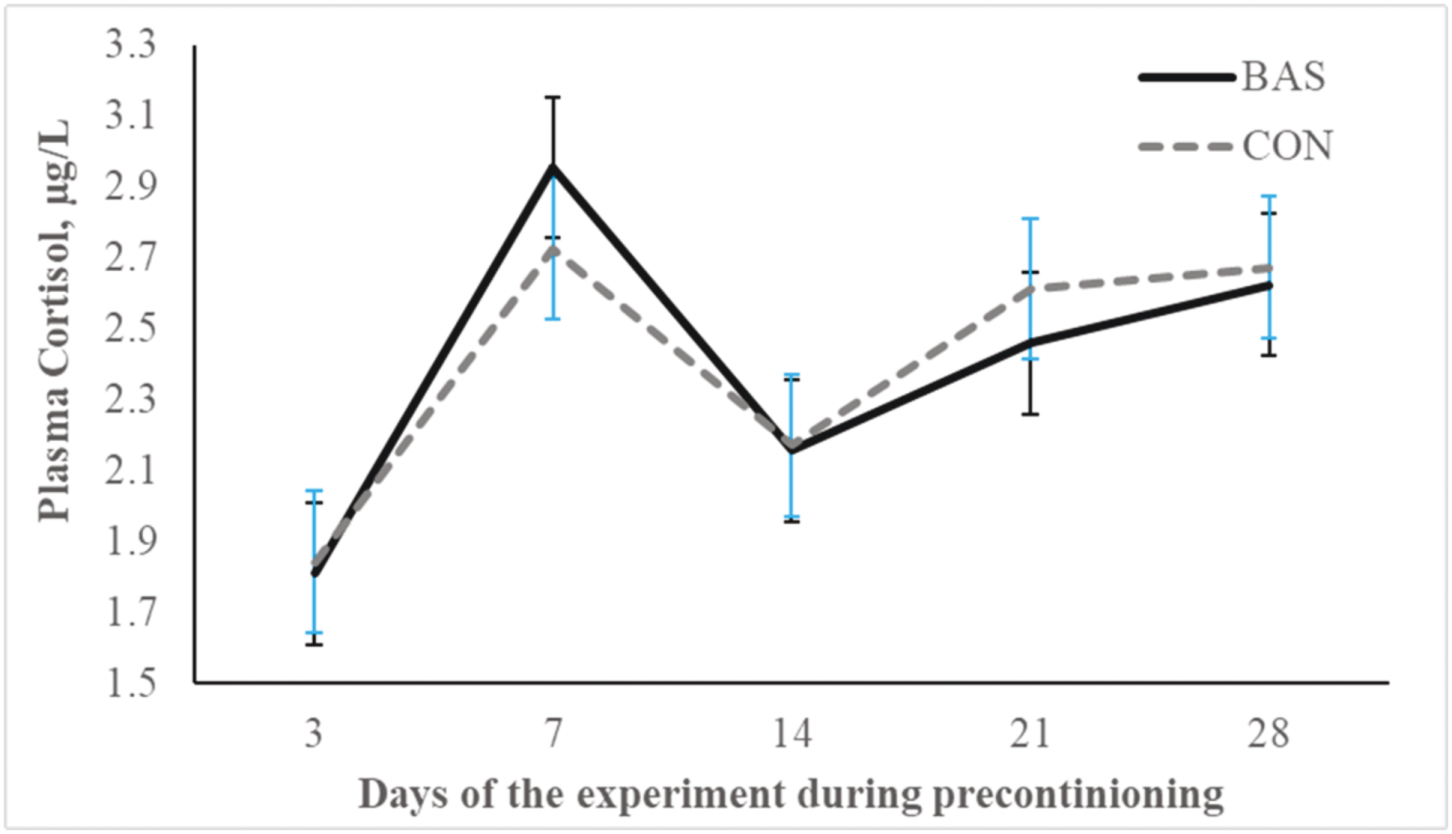
Preconditioning phase: plasma cortisol concentration of beef calves administered multiple bovine-appeasing substance (**BAS,***n* = 9) or not (**CON**, *n* = 9) every 14 d during a 42-d preconditioning followed by a feedlot receiving phase (days 43 to 90). Calves individually received 5 mL of a BAS (IRSEA Group, Quartier Salignan, France) or CON (diethylene glycol monoethyl ether) topically to their nuchal skin area on days 0, 14, 28, and 42. Values obtained from day 0 were used as an independent covariate. Therefore, the results reported are covariate-adjusted least square means using the pen as the experimental unit (*n* = 9/treatment), and the treatment effect was tested with 16 °C of freedom. A treatment × day interaction was not detected (*P* = 0.81).

**Figure 3.**
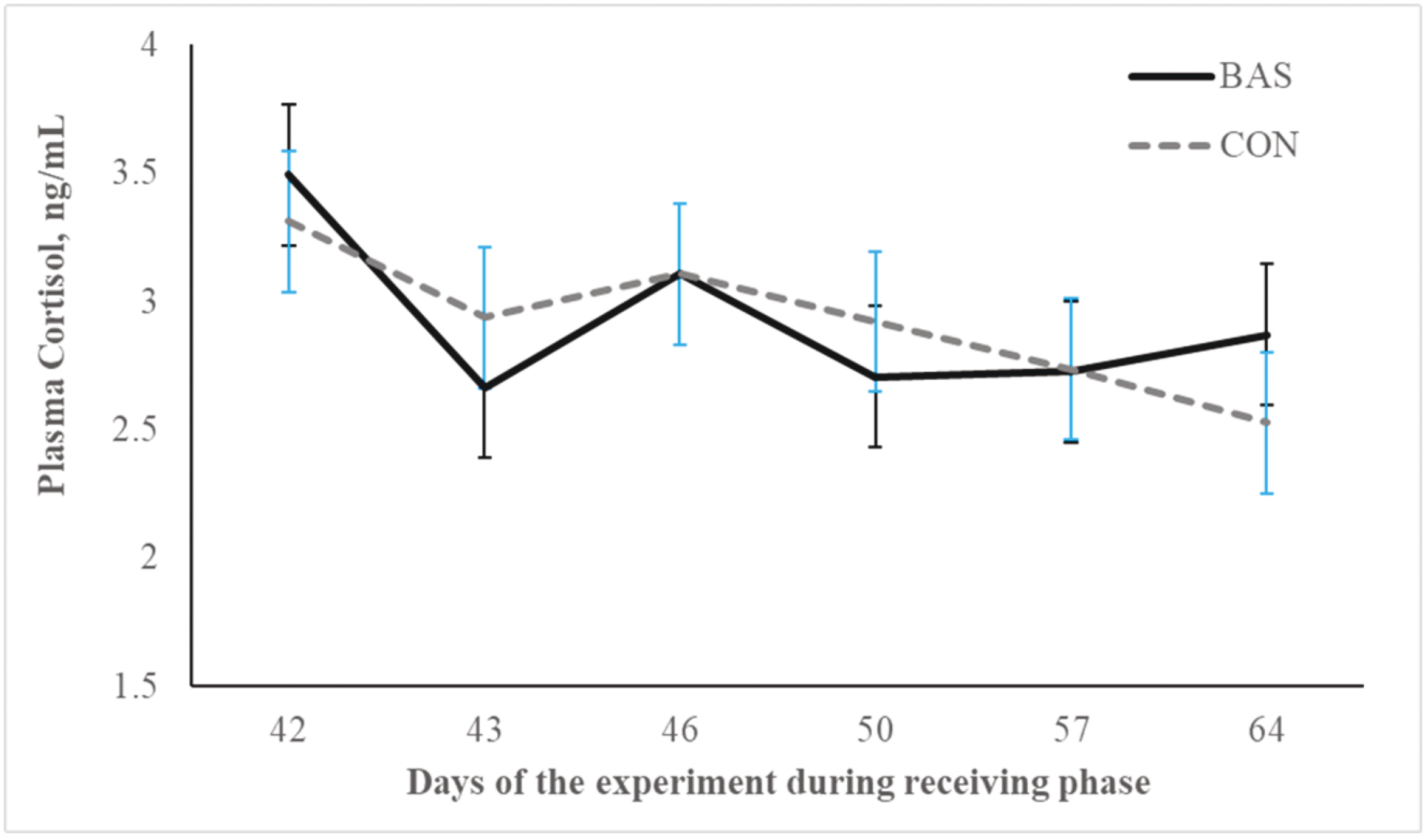
Receiving phase: plasma cortisol concentration of beef calves administered multiple bovine-appeasing substance (**BAS,***n* = 9) or not (**CON**, *n* = 9) every 14 d during a 42-d preconditioning followed by a feedlot receiving phase (days 43 to 90). Calves individually received 5 mL of a BAS (IRSEA Group, Quartier Salignan, France) or CON (diethylene glycol monoethyl ether) topically to their nuchal skin area on days 0, 14, 28, and 42. Values obtained from day 0 were used as an independent covariate. Therefore, the results reported are covariate-adjusted least square means using the pen as the experimental unit (*n* = 9/treatment), and the treatment effect was tested with 16 °C of freedom. A treatment × day interaction was not detected (*P* = 0.58).

**Figure 4.**
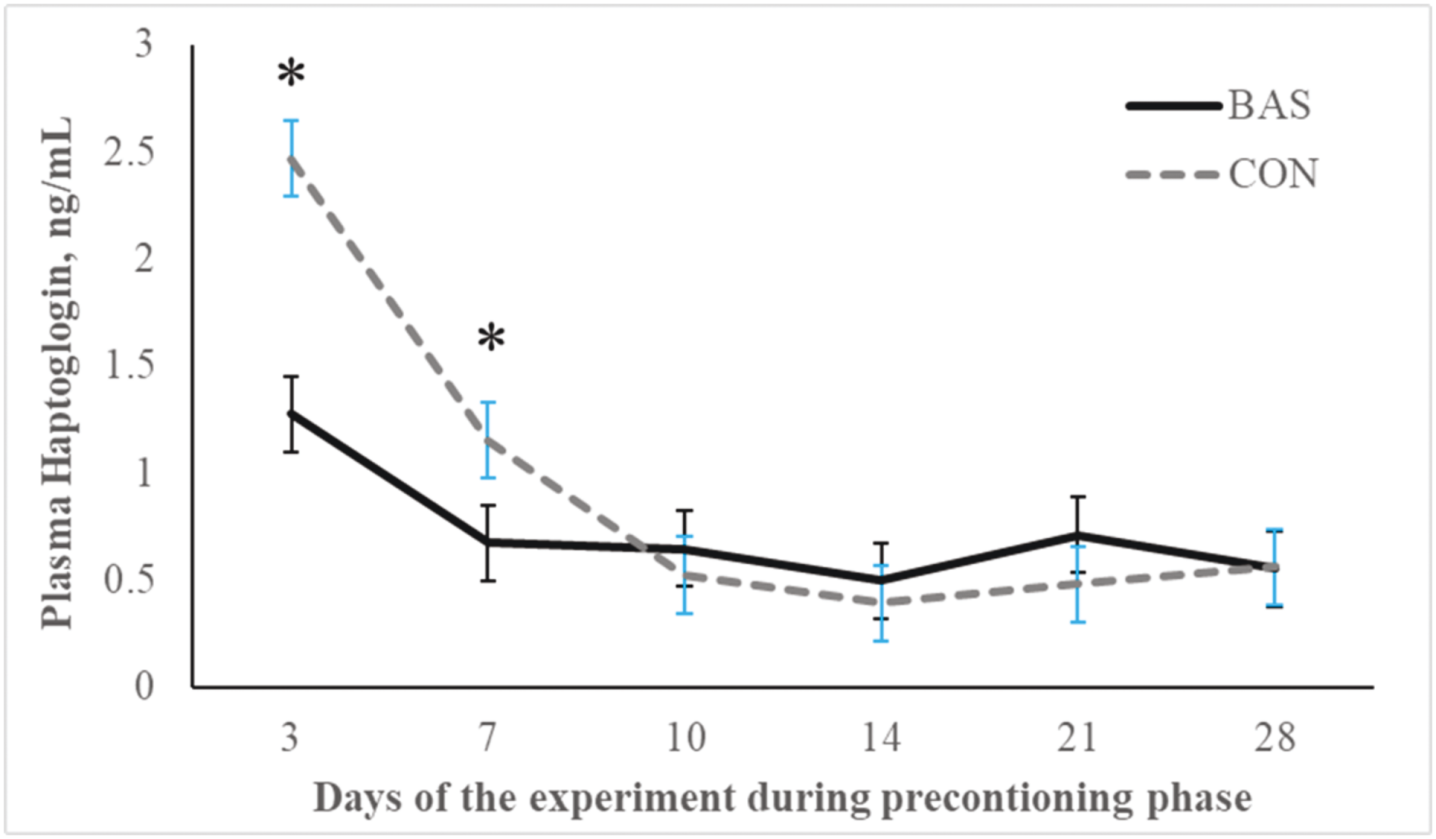
Preconditioning phase: plasma haptoglobin concentration of beef calves administered multiple bovine-appeasing substances (**BAS,***n* = 9) or not (**CON**, *n* = 9) every 14 d during a 42-d preconditioning followed by a feedlot receiving phase (days 43 to 90). Calves individually received 5 mL of a BAS (IRSEA Group, Quartier Salignan, France) or CON (diethylene glycol monoethyl ether) topically to their nuchal skin area on days 0, 14, 28, and 42. Values obtained from day 0 were used as an independent covariate. Therefore, the results reported are covariate-adjusted least square means using the pen as the experimental unit (*n* = 9/treatment), and the treatment effect was tested with 16 °C of freedom. A treatment × day interaction was detected (*P* = 0.001). Within day, *P < 0.01.

**Figure 5.**
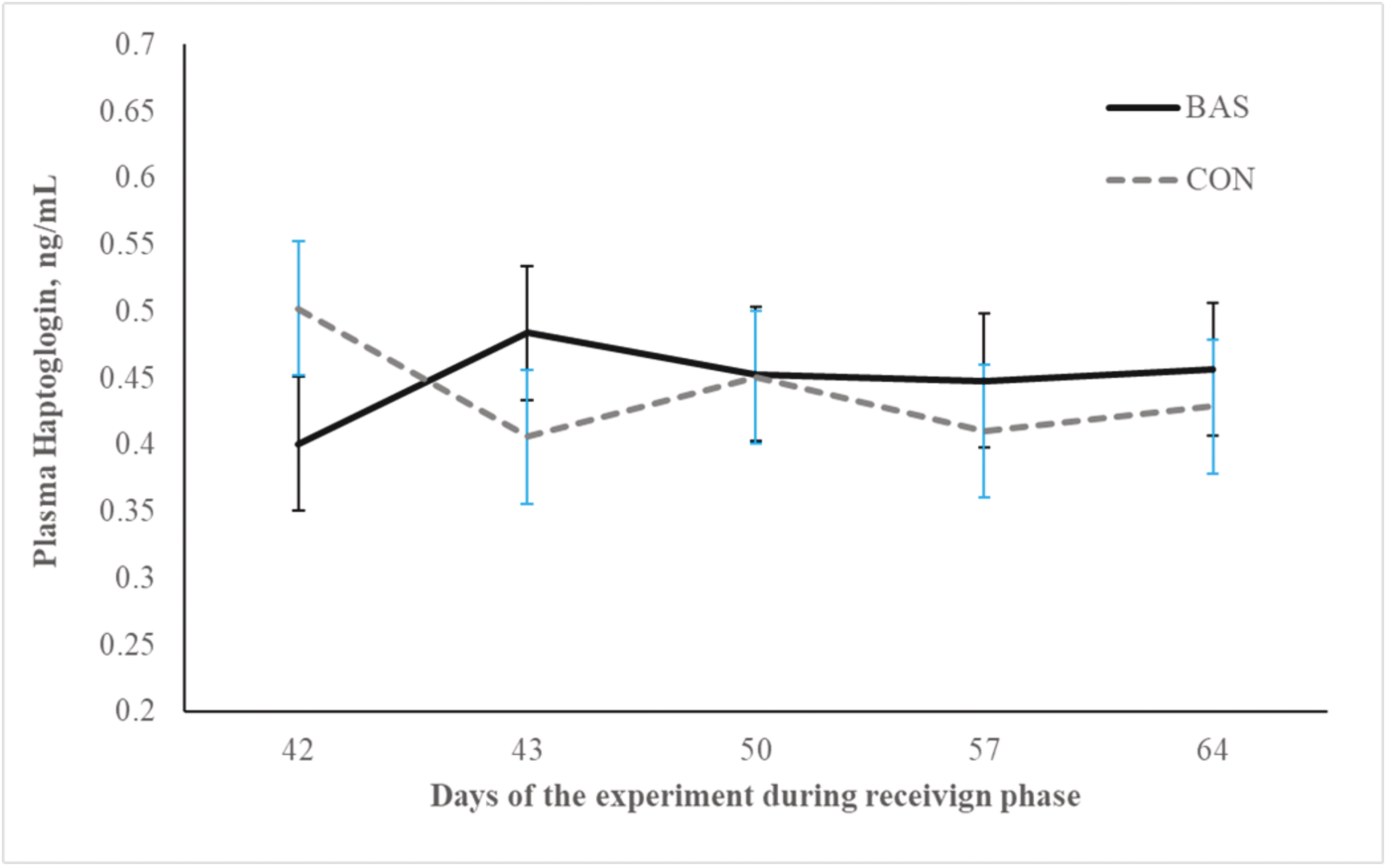
Receiving phase: plasma haptoglobin concentration of beef calves administered multiple bovine appeasing substances (**BAS,***n* = 9) or not (**CON**, *n* = 9) every 14 d during a 42-d preconditioning followed by a feedlot receiving phase (days 43 to 90). Calves individually received 5 mL of a BAS (IRSEA Group, Quartier Salignan, France) or CON (diethylene glycol monoethyl ether) topically to their nuchal skin area on days 0, 14, 28, and 42. Values obtained from day 0 were used as an independent covariate. Therefore, the results reported are covariate-adjusted least square means using the pen as the experimental unit (*n* = 9/treatment), and the treatment effect was tested with 16 °C of freedom. A treatment × day interaction was not detected (*P* = 0.64).

**Figure 6.**
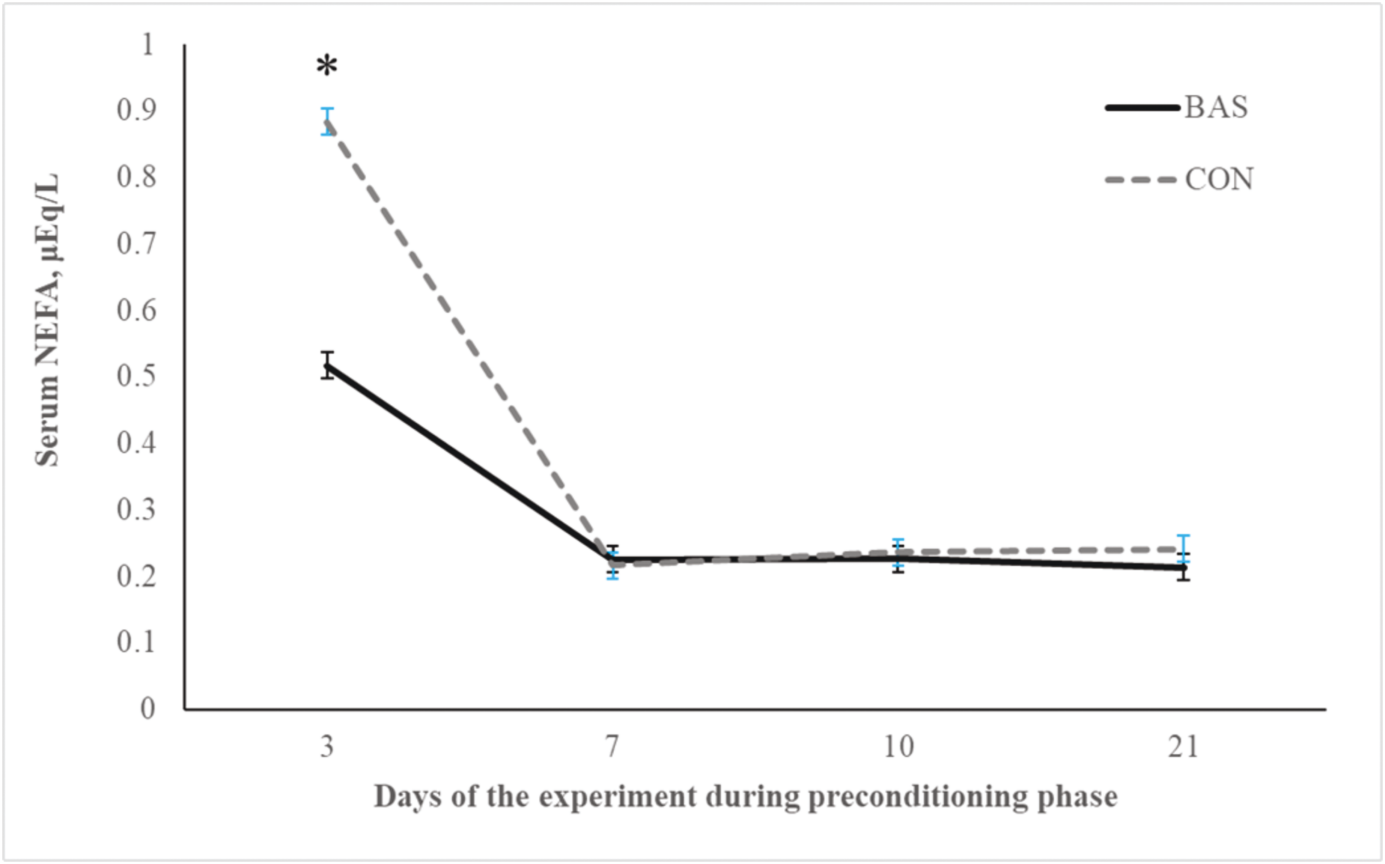
Preconditioning phase: serum NEFA concentration of beef calves administered multiple bovine appeasing substances (**BAS,***n* = 9) or not (**CON**, *n* = 9) every 14 d during a 42-d preconditioning followed by a feedlot receiving phase (days 43 to 90). Calves individually received 5 mL of a BAS (IRSEA Group, Quartier Salignan, France) or CON (diethylene glycol monoethyl ether) topically to their nuchal skin area on days 0, 14, 28, and 42. Values obtained from day 0 were used as an independent covariate. Therefore, the results reported are covariate-adjusted least square means using the pen as the experimental unit (*n* = 9/treatment), and the treatment effect was tested with 16 °C of freedom. A treatment × day interaction was detected (*P* = 0.001). Within day, *P < 0.01.

**Figure 7.**
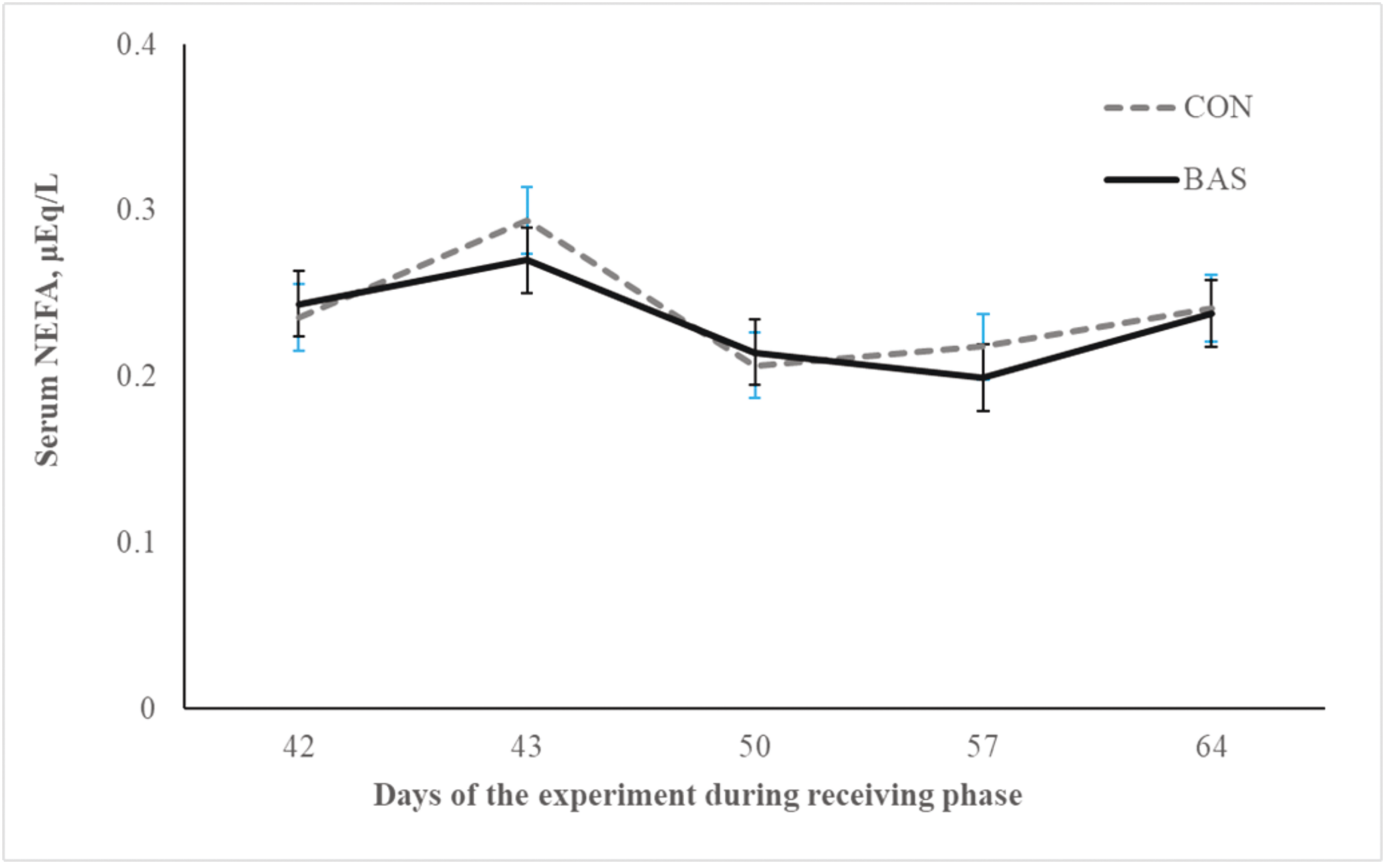
Receiving phase: serum NEFA concentration of beef calves administered multiple bovine appeasing substances (**BAS,***n* = 9) or not (**CON**, *n* = 9) every 14 d during a 42-d preconditioning followed by a feedlot receiving phase (days 43 to 90). Calves individually received 5 mL of a BAS (IRSEA Group, Quartier Salignan, France) or CON (diethylene glycol monoethyl ether) topically to their nuchal skin area on days 0, 14, 28, and 42. Values obtained from day 0 were used as an independent covariate. Therefore, the results reported are covariate-adjusted least square means using the pen as the experimental unit (*n* = 9/treatment), and the treatment effect was tested with 16 °C of freedom. A treatment × day interaction was not detected (*P* = 0.85).

**Figure 8.**
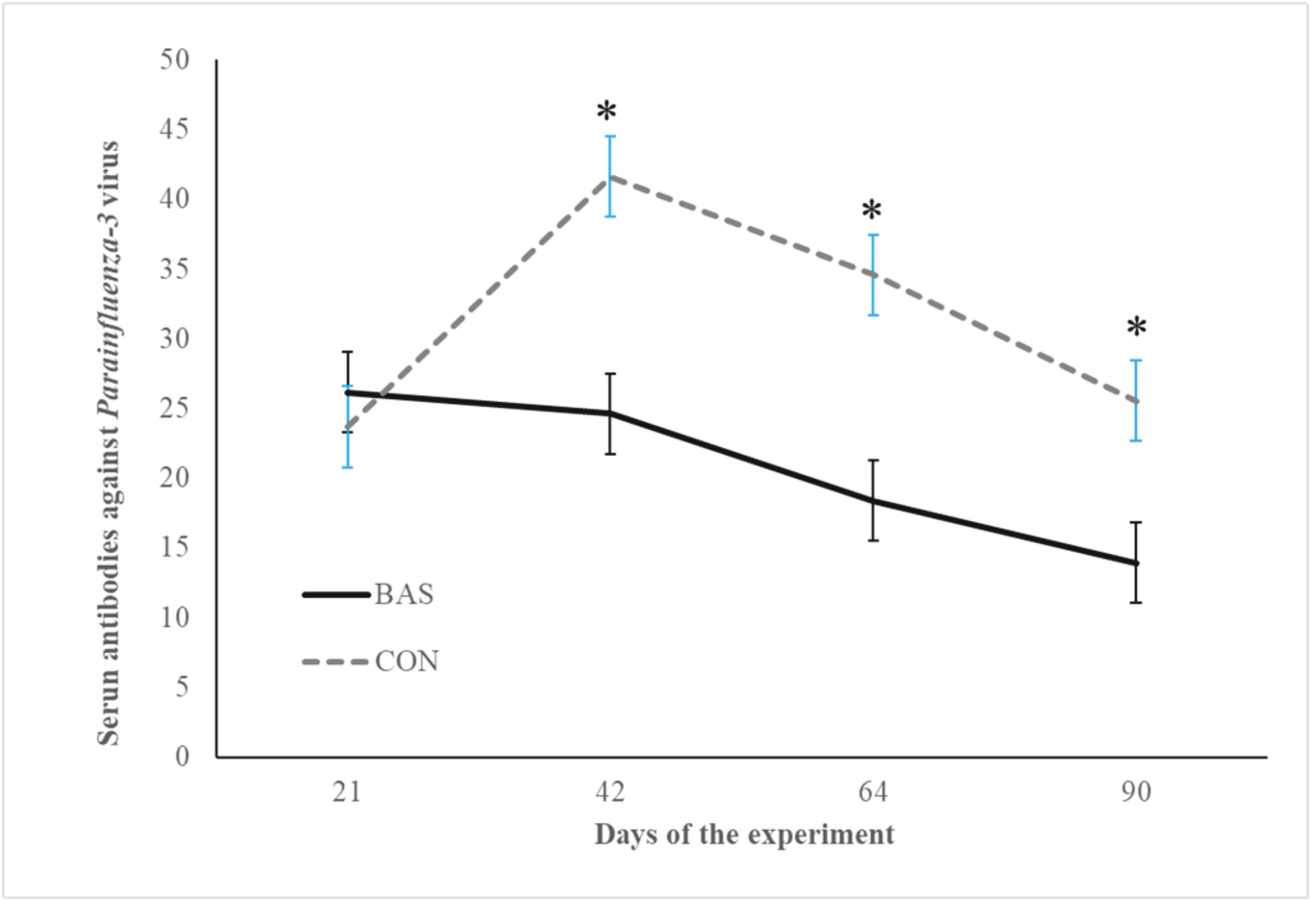
Serum concentration of antibody against parainfluenza-3 virus (PI-3) of beef calves administered multiple bovine appeasing substances (**BAS,***n* = 9) or not (**CON**, *n* = 9) every 14 d during a 42-d preconditioning followed by a feedlot receiving phase (days 43 to 90). Calves individually received 5 mL of a BAS (IRSEA Group, Quartier Salignan, France) or CON (diethylene glycol monoethyl ether) topically to their nuchal skin area on days 0, 14, 28, and 42. On day 0, calves were vaccinated against *Clostridium* and *Mannheimia haemolytica* (One Shot Ultra 7; Zoetis Florham Park, NJ), *infectious bovine rhinotracheitis virus*, *bovine viral diarrhea complex, parainfluenza-3 virus, and bovine respiratory syncytial virus* (Bovi-Shield Gold 5; Zoetis), and were administered an anthelmintic (Dectomax; Zoetis). On day 21, calves were re-vaccinated against *Clostridium* (Ultrabac 8; Zoetis), *infectious bovine rhinotracheitis virus, bovine viral diarrhea complex, parainfluenza-3, and bovine respiratory syncytial virus* (Bovi-Shield Gold 5; Zoetis). Values obtained from day 0 were used as an independent covariate within each experimental period. Therefore, the results reported are covariately adjusted least square means using the pen as the experimental unit (*n* = 9/treatment), and the treatment effect was tested with 16 °C of freedom. A treatment × day interaction was detected (*P* = 0.001). Within day, *P < 0.01.

**Figure 9.**
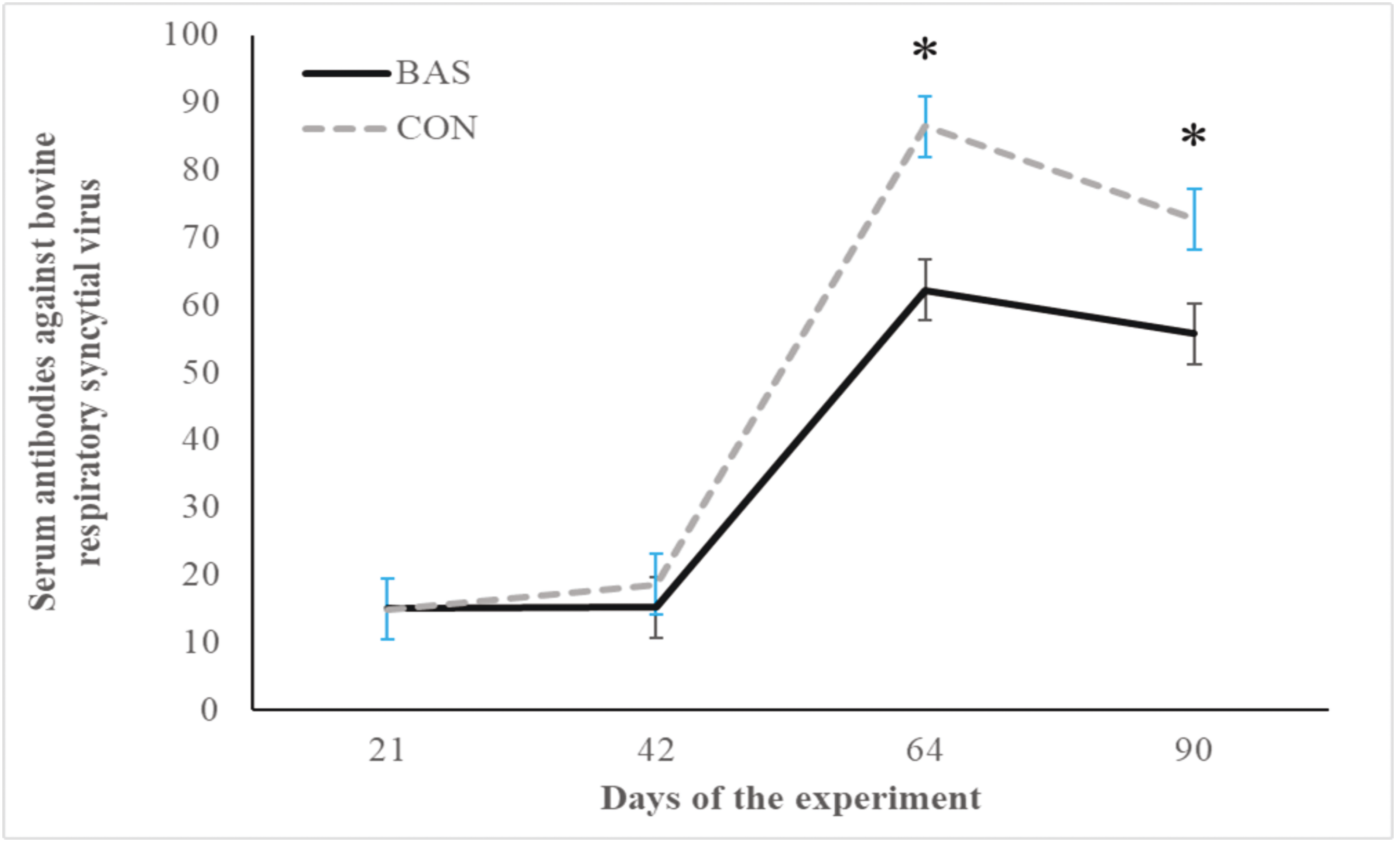
Serum concentration of antibody against bovine respiratory syncytial virus (BRSV) of beef calves administrated multiple bovine appeasing substances (**BAS,***n* = 9) or not (**CON**, *n* = 9) every 14 d during a 42-d preconditioning followed by a feedlot receiving phase (days 43 to 90). Calves individually received 5 mL of a BAS (IRSEA Group, Quartier Salignan, France) or CON (diethylene glycol monoethyl ether) topically to their nuchal skin area on days 0, 14, 28, and 42. On day 0, calves were vaccinated against *Clostridium* and *Mannheimia haemolytica* (One Shot Ultra 7; Zoetis Florham Park, NJ), *infectious bovine rhinotracheitis virus*, *bovine viral diarrhea complex, parainfluenza-3 virus, and bovine respiratory syncytial virus* (Bovi-Shield Gold 5; Zoetis), and were administered an anthelmintic (Dectomax; Zoetis). On day 21, calves were re-vaccinated against *Clostridium* (Ultrabac 8; Zoetis), *infectious bovine rhinotracheitis virus, bovine viral diarrhea complex, parainfluenza-3, and bovine respiratory syncytial virus* (Bovi-Shield Gold 5; Zoetis). Values obtained from day 0 were used as an independent covariate within each experimental period. Therefore, the results reported are covariately adjusted least square means using the pen as the experimental unit (*n* = 9/treatment), and the treatment effect was tested with 16 °C of freedom. A treatment × day interaction was detected (*P* = 0.001). Within day, *P < 0.01.

**Figure 10.**
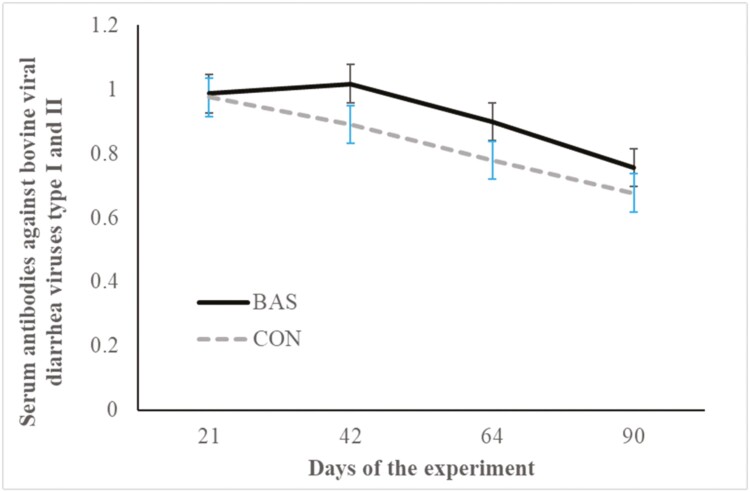
Serum concentration of antibody against bovine viral diarrhea viruses I and II (BVDV) of beef calves administrated multiple bovine appeasing substance (**BAS,***n* = 9) or not (**CON**, n = 9) every 14 d during a 42-d preconditioning followed by a feedlot receiving phase (days 43 to 90). Calves individually received 5 mL of a BAS (IRSEA Group, Quartier Salignan, France) or CON (diethylene glycol monoethyl ether) topically to their nuchal skin area on days 0, 14, 28, and 42. On day 0, calves were vaccinated against *Clostridium* and *Mannheimia haemolytica* (One Shot Ultra 7; Zoetis Florham Park, NJ), *infectious bovine rhinotracheitis virus*, *bovine viral diarrhea complex, parainfluenza-3 virus, and bovine respiratory syncytial virus* (Bovi-Shield Gold 5; Zoetis), and were administered an anthelmintic (Dectomax; Zoetis). On day 21, calves were re-vaccinated against *Clostridium* (Ultrabac 8; Zoetis), *infectious bovine rhinotracheitis virus, bovine viral diarrhea complex, parainfluenza-3, and bovine respiratory syncytial virus* (Bovi-Shield Gold 5; Zoetis). Values obtained from day 0 were used as an independent covariate within each experimental period. Therefore, the results reported are covariately adjusted least square means using the pen as the experimental unit (*n* = 9/treatment), and the treatment effect was tested with 16 °C of freedom. A treatment × day interaction was not detected (*P* = 0.26).

**Figure 11.**
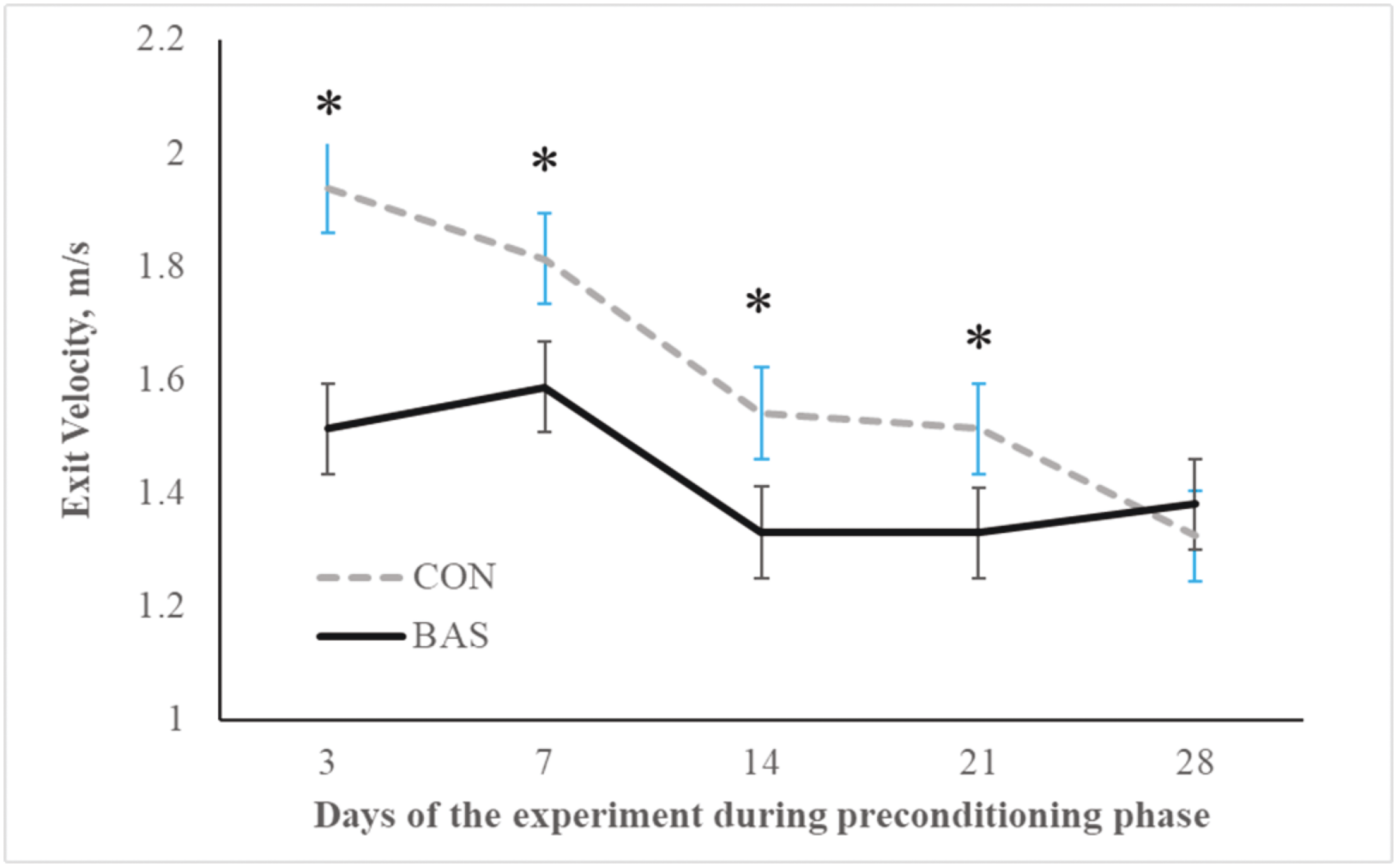
Preconditioning phase: Exit velocity of beef calves administered multiple bovine appeasing substances (**BAS,***n* = 9) or not (**CON**, *n* = 9) every 14 d during a 42-d preconditioning followed by a feedlot receiving phase (days 43 to 90). Calves individually received 5 mL of a BAS (IRSEA Group, Quartier Salignan, France) or CON (diethylene glycol monoethyl ether) topically to their nuchal skin area on days 0, 14, 28, and 42. Values obtained from day 0 were used as an independent covariate. Therefore, the results reported are covariate-adjusted least square means using the pen as the experimental unit (*n* = 9/treatment), and the treatment effect was tested with 16 °C of freedom. A treatment × day interaction was detected (*P* = 0.001). Within day, *P < 0.01.

**Figure 12.**
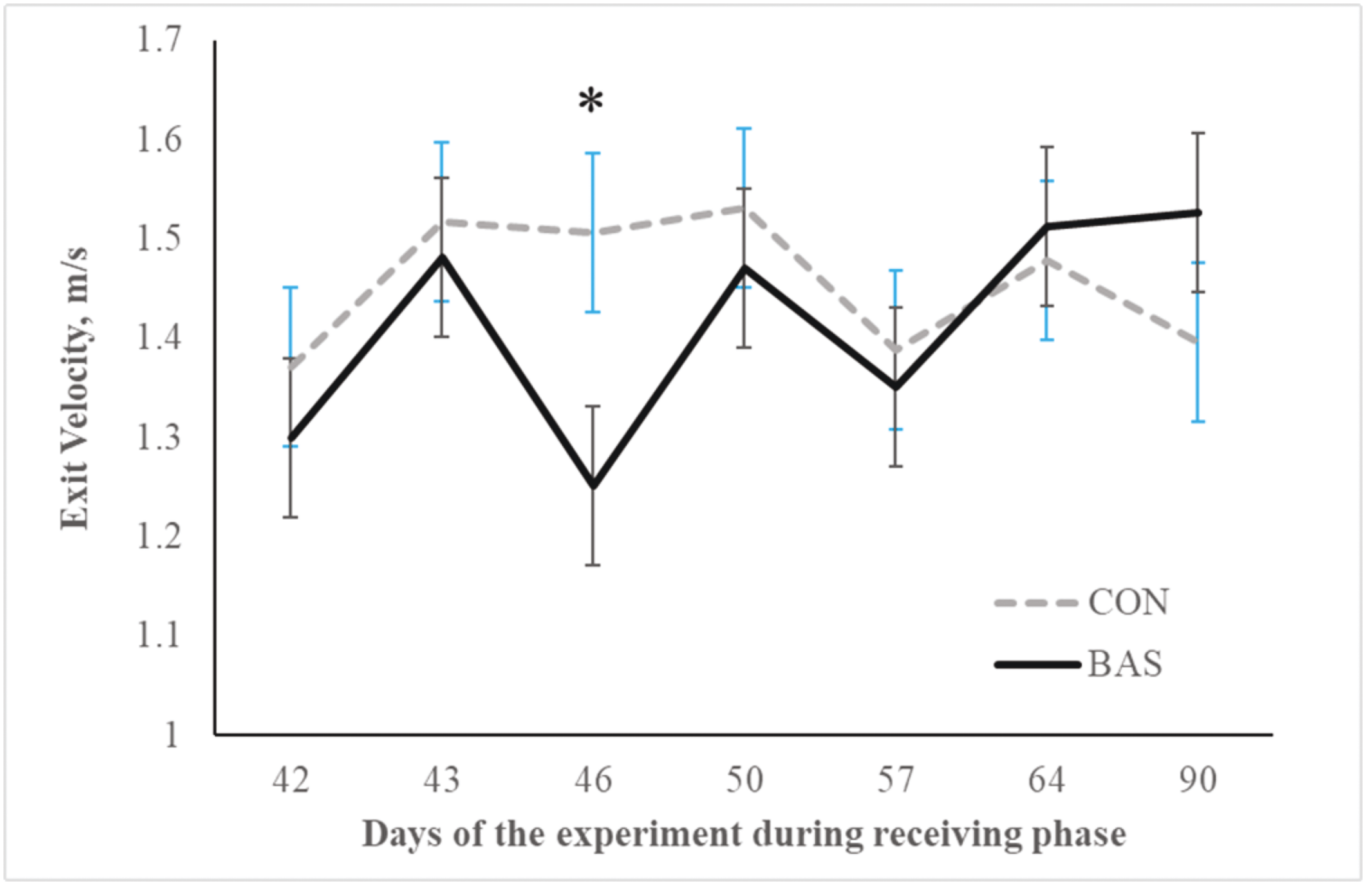
Receiving phase: exit velocity of beef calves administered multiple bovine appeasing substances (**BAS,***n* = 9) or not (**CON**, *n* = 9) every 14 d during a 42-d preconditioning followed by a feedlot receiving phase (days 43 to 90). Calves individually received 5 mL of a BAS (IRSEA Group, Quartier Salignan, France) or CON (diethylene glycol monoethyl ether) topically to their nuchal skin area on days 0, 14, 28, and 42. Values obtained from day 0 were used as an independent covariate. Therefore, the results reported are covariate-adjusted least square means using the pen as the experimental unit (*n* = 9/treatment), and the treatment effect was tested with 16 °C of freedom. A treatment × day interaction was detected (*P* = 0.001). Within a day, *P < 0.01.

No treatment effects were detected (*P* = 0.98) for plasma concentrations of cortisol (Figures 2 and 3) during the preconditioning and receiving phases. A treatment × day interaction was detected (*P* < 0.001) for plasma haptoglobin concentrations during the preconditioning phase (Figure 4). Plasma haptoglobin concentration was greater (*P* < 0.01) in CON calves on days 3 and 7 compared with BAS calves. During the receiving phase, however, plasma haptoglobin concentration was not impacted (*P *> 0.62) by treatments (Figure 5). A treatment × day interaction was detected (*P* < 0.001) for serum NEFA concentration during the preconditioning phase (Figure 6) but not (*P *> 0.85) during the receiving phase (Figure 7). During the preconditioning phase, serum NEFA concentration was reduced (*P* < 0.01) in BAS on day 3 compared with CON calves. Calves that received BAS had decreased (*P* < 0.001) serum concentrations of antibodies against PI-3 (Figure 8) and BRSV (Figure 9) during the experiment compared with CON calves (Table 3). However, no treatment effects were noted (*P *= 0.15) for serum antibody concentrations against BVDV type I and II, whereas day effects were detected (*P* < 0.01) for all serum variables (Table 3; Figure 10). A treatment × day interaction was detected (*P* = 0.001) for exit velocity, which was greater (*P* < 0.001) for CON vs. BAS calves on days 3, 7, 14, and 21 during the preconditioning phase (Figure 11) and on day 46 of the receiving phase (Figure 12).

**Table 3.** Physiological responses and exit velocity of beef calves administered multiple bovine appeasing (**BAS,***n* = 9) or not (**CON**, *n* = 9) every 14 d during a 42-d preconditioning phase followed by a feedlot receiving phase (days 43 to 90)^1^

				*P-*value		
Item	CON	BAS	SEM	Treatment	Day	Treament × day
*Preconditioning phase* ^2^						
Plasma cortisol, ng/mL	2.41	2.40	0.14	0.98	<0.001	0.81
Plasma haptoglobin, ng/mL	0.51	0.50	0.02	0.72	<0.001	0.001
Serum NEFA, μEq/L	0.22	0.36	0.01	<0.001	<0.001	<0.001
Exit velocity, m/s	1.63	1.42	0.06	0.01	<0.001	0.03
*Receiving phase* ^2^						
Plasma cortisol, µg/L	2.78	2.84	0.21	0.82	0.28	0.58
Plasma haptoglobin, ng/mL	0.42	0.41	0.02	0.62	0.89	0.64
Serum NEFA, μEq/L	0.26	0.23	0.01	0.46	<0.001	0.85
Exit velocity, m/s	1.48	1.35	0.04	0.01	0.02	0.05
*Serum antibodies against respiratory viruses* ^3^						
Parainfluenza-3 virus	31.36	20.77	2.0	<0.001	<0.001	<0.001
Bovine respiratory syncytial virus	51.12	40.32	3.6	0.04	<0.001	<0.001
Bovine viral diarrhea viruses type I and II	0.838	0.968	0.06	0.15	<0.001	0.26

^1^Calves individually received 5 mL of a BAS (IRSEA Group, Quartier Salignan, France) or CON (diethylene glycol monoethyl ether) topically to their nuchal skin area on days 0, 14, 28, and 42. On day 42, calves were combined within the treatment group, loaded into 2 different single double-deck commercial livestock trailers, and transported for 1,000 km (approximately 16 h). Upon arrival (day 43), calves were unloaded at the same feedyard and with the same pen distribution used before transport but allocated to different drylot pens. From days 43 to 90, calves were fed ad libitum a TMR diet based on the same ingredients used during the preconditioning program, whereas transitioned using a multiple-step-up diet program. Pen was the experimental unit (*n* = 9/treatment). Therefore, the treatment effect was tested with 16 °C of freedom.

^2^Blood samples were collected on days 0 (weaning), 3, 7, 14, 21, 28, 42 (prior transport), 43 (feedlot arrival), 46, 50, 57, 64, and 90 via jugular venipuncture. Results from day 0 were used as covariates in each respective analysis.

^3^On day 0, calves were vaccinated against Clostridium and Mannheimia haemolytica (One Shot Ultra 7; Zoetis Florham Park, NJ), infectious bovine rhinotracheitis virus, bovine viral diarrhea complex, parainfluenza-3 virus, and bovine respiratory syncytial virus (Bovi-Shield Gold 5; Zoetis) and were administered an anthelmintic (Dectomax; Zoetis). On day 21, calves were re-vaccinated against Clostridium (Ultrabac 8; Zoetis), infectious bovine rhinotracheitis virus, bovine viral diarrhea complex, parainfluenza-3, and bovine respiratory syncytial virus (Bovi-Shield Gold 5; Zoetis). Serum samples collected on days 0, 21, 42, and 64 were analyzed for antibodies against the respiratory virus and results were expressed as sample:positive control ratio. Results from day 0 were used as covariates in each respective analysis.

## Discussion

Transitioning from weaning to entering the feedlot instigates many stressors for the calf, including separation from the dam, dietary shifts, and a reshaped social environment (Weary et al., 2008). Moreover, weaning is often compounded with additional stressors such as transportation, vaccinations, encounters with unfamiliar humans, and exposure to new environments (Marques et al., 2012; Cooke, 2017), further intensifying the overall stress experience. These stressful events are known to stimulate an inflammatory response, neuroendocrine activation, and mobilization of reserves that are negatively associated with cattle performance in beef cattle systems (Marques et al., 2012, 2019; Cooke, 2017). Although cortisol concentrations evaluated herein did not differ between treatments, a day effect was noted for plasma cortisol concentration, which corroborates that calves experienced a hypothalamic-pituitary-adrenal response during the preconditioning phase. In addition, our study showed that calves herein experienced an inflammatory reaction elicited by the combination of weaning, vaccination, and novel management during the preconditioning phase (Cooke, 2017; Marques et al., 2019; Abreu et al., 2024). Administering BAS to recently weaned calves in the present study decreased haptoglobin and NEFA concentrations, indicating and corroborating previous reports that BAS alleviates an acute-phase reaction and decreases mobilization of body tissues during a stress event (Cappellozza et al., 2020; Colombo et al., 2020; Cooke et al., 2020; Schubach et al., 2020). Accordingly, a recent study by Hervet et al. (2021) demonstrated that bulls administrated BAS had decreased blood mRNA expression of genes linked to pro-inflammatory processes after weaning and feedlot entry. According to the manufacturer, the BAS is estimated to remain in the animals for approximately 15 d after administration, which corroborates with the benefits on calf performance and the immune response observed by Cooke et al. (2020). Osella et al. (2018) administered BAS to dairy cows weekly upon turn out to pasture and reported productive benefits throughout their 28-d experimental period. Nonetheless, multiple administration of BAS in our study did not alter the nutritional and metabolic status of the calves during the receiving phase.

Psychological and physical stress experienced by the calves due to weaning and transportation might increase agitation or aggressivity responses when exposed to human handling, activating adrenocortical and acute-phase protein responses in cattle (Francisco et al., 2012; Cooke, 2014). Pheromones are substances secreted by one animal that influence the physiology or behavior of another animal. The postulated mechanism of these results could be explained by the actions of these substances on the olfactory epithelium or vomeronasal organ of mammals (Holy et al., 2000). Stimulation of these sites by a biologically relevant chemical signal might significantly affect livestock animals’ behavior and physiology (McGlone and Anderson, 2002; Archunan et al., 2014). Accordingly, the application of a synthetic analog to the endogenous substance secreted by the sow skin reduced piglets aggressive biting and antagonistic behavior and increased time feeding and standing/walking and performance (Pageat and Teyssier, 1998; Pageat, 2001; McGlone and Anderson, 2002). Hence, bovine-appeasing substance administration was expected to impact these responses due to its calming effects (Archunan et al., 2014). In the present study, multiple administrations of BAS every 14 d reduced the exit velocity of the calves on days 3, 7, 14, and 21 during the preconditioning phase and day 46 during the receiving phase, indicating that BAS might increase the ability of the calves to cope with the stress of routine management practices (i.e., weaning and transportation), by reducing aggressivity and inflammatory responses (Cooke, 2014; Schubach et al., 2020). Accordingly, Schubach et al. (2020) also demonstrated a reduced exit velocity of BAS calves on days 7 and 14 after weaning, which might be attributed to the reduced overall stress response.

Calves in this study effectively developed humoral immunity against BRD pathogens through vaccination at weaning and a booster administered 21 d later. However, contrary to previous findings (Colombo et al., 2020; Schubach et al., 2020; Vieira et al., 2023), an unexpected result emerged: calves from the CON group exhibited greater acquired immunity to BRD pathogens than those from the BAS group. Previous research suggests that a more consistent immune function, characterized by reduced inflammatory responses, enhances the effectiveness of vaccination (Munck et al., 1984; Biolatti et al., 2005; Vieira et al., 2023). Accordingly, Schubach et al. (2020) attributed the benefits of acquired immunity against BRD to the diminished adrenocortical and acute-phase responses observed in calves that received BAS. Despite the differences observed in inflammatory responses in this study for calves administrated BAS, these outcomes did not significantly impact the acquired humoral immunity of BAS compared with CON calves. One could speculate that multiple administrations of BAS might cause a diminished response of the acquired humoral against BRD pathogens compared to CON, which deserves further investigation.

Overall ADG, TMR intake, and G:F during the preconditioning and receiving phase were not impacted by multiple applications of BAS. Contrary, previous research with BAS (Cappellozza et al., 2020; Colombo et al., 2020; Cooke et al., 2020; Fonseca et al., 2021; Vieira et al., 2023; Cappellozza et al., 2020; Cooke et al., 2020) have shown immediate benefits on performance of calves administrated BAS. In line with this, recent studies have highlighted that administering BAS to beef calves at weaning and to beef bulls upon arrival at the feedlot enhances initial BW gain (Cappellozza et al., 2020; Colombo et al., 2020; Cooke et al., 2020; Fonseca et al., 2021). Additionally, Cappellozza et al. (2020) found that BAS administration to beef calves at weaning improved both ADG and final BW over a 45-d preconditioning period. It is important to note that in Cappelloza et al. (2020), Cooke et al. (2020), Colombo et al. (2020), and Fonseca et al. (2021), the sampling frequency (every 7 to 19 d) was less intense than the present study, which could partially explain the lack of difference in performance responses, disrupting and hindering the full benefits of BAS. Corroborating this rationale, multiple applications of BAS resulted in a numerical difference in the final BW of the calves at the end of the receiving period, where the number of samplings was reduced, which also could explain the difference in IGF-1 concentration, indicating an enhanced nutritional status of BAS calves at the end of the receiving phase. Hence, multiple administration of a bovine-appeasing substance during the preconditioning period appear to have contributed to reduced acute-phase responses and exit velocity, indicating that BAS could be used as a management strategy to promote healthy responses to recently weaning calves. Despite the lack of differences in the performance, additional research is warranted to evaluate the potential carryover benefits of BAS throughout growing and finishing phase due to the numerical increase in BW and IGF-1 concentration by the end of the trial.

## Abbreviations

ADG: average daily gain
BAS: bovine-appeasing substance
BRS: bovine respiratory disease
BW: body weight
DMI: dry matter intake
G:F: feed efficiency
TMR: total mixed ration.

## References

[CIT0001] Abreu, M. J. I., R. S. Marques, I. A. Cidrini, L. H. C. Batista, I. M. Ferreira, K. A. Oliveira, V. A. Cruz, A. C. Limede, L. M. Sousa, M. Q. S. França, G. H. M. Bísio, G. R. Siqueira, and F. D. Resende. 2024. Long-term impacts of 48-h water and feed deprivation on blood and performance responses of grazing Bos indicus Nellore heifers. Transl Anim Sci. 8:txae015. doi: 10.1093/tas/txae015 38371423 PMC10872672

[CIT0032] AOAC. 2006. Official methods of analysis. 18th ed. AOAC Int., Arlington, VA.

[CIT0002] Archunan, G., S. Rajanarayanan, and K. Karthikeyan. 2014. Cattle Pheromones. In: C. Mucignat-Caretta, editor. Neurobiology of Chemical Communication. Boca Raton, FL: CRC Press/Taylor & Francis. http://www.ncbi.nlm.nih.gov/books/NBK200988/24830034

[CIT0004] Biolatti, B., E. Bollo, F. T. Cannizzo, G. Zancanaro, M. Tarantola, M. Dacasto, M. Cantiello, M. Carletti, P. G. Biolatti, and G. Barbarino. 2005. Effects of low-dose dexamethasone on thymus morphology and immunological parameters in veal calves. J. Vet. Med. A Physiol. Pathol. Clin. Med. 52:202–208. doi: 10.1111/j.1439-0442.2005.00714.x 15882406

[CIT0005] Cappellozza, B. I., J. P. Bastos, and R. F. Cooke. 2020. Short communication: Administration of an appeasing substance to Bos indicus-influenced beef cattle improves performance after weaning and carcass pH. Livest. Sci. 238:104067. doi: 10.1016/j.livsci.2020.104067

[CIT0007] Colombo, E. A., R. F. Cooke, A. P. Brandão, J. B. Wiegand, K. M. Schubach, G. C. Duff, V. N. Gouvêa, and B. I. Cappellozza. 2020. Administering an appeasing substance to optimize performance and health responses in feedlot receiving cattle. J. Anim. Sci. 98:1–8. doi: 10.1093/jas/skaa339. PMC7648383 33068399

[CIT0008] Cooke, R. F. 2014. Temperament and acclimation to human handling influence growth, health, and reproductive responses in Bos taurus and B. indicus cattle. J. Anim. Sci. 92:5325–5333. doi: 10.2527/jas.2014-8017 25023802

[CIT0009] Cooke, R. F. 2017. Invited Paper: nutritional and management considerations for beef cattle experiencing stress-induced inflammation 1 1This article was based on a presentation at the ARPAS Symposium “Understanding Inflammation and Inflammatory Biomarkers to Improve Animal Performance” at the 2016 Joint Annual Meeting, July 19–23, 2016, Salt Lake City, Utah. Prof. Anim. Sci. 33:1–11. doi: 10.15232/pas.2016-01573

[CIT0010] Cooke, R. F., and J. D. Arthington. 2013. Concentrations of haptoglobin in bovine plasma determined by ELISA or a colorimetric method based on peroxidase activity: Methods to determine haptoglobin in bovine plasma. J. Anim. Physiol. Anim. Nutr. (Berl) 97:531–536. doi: 10.1111/j.1439-0396.2012.01298.x 22487219

[CIT0011] Cooke, R. F., A. Millican, A. P. Brandão, T. F. Schumaher, O. A. de Sousa, T. Castro, R. S. Farias, and B. I. Cappellozza. 2020. Short communication: administering an appeasing substance to Bos indicus -influenced beef cattle at weaning and feedlot entry. Animal. 14:566–569. doi: 10.1017/S1751731119002490 31635563

[CIT0012] Duff, G. C., and M. L. Galyean. 2006. BOARD-INVITED REVIEW: recent advances in management of highly stressed, newly received feedlot cattle. J. Anim. Sci. 85:823–840. doi: 10.2527/jas.2006-501 17085724 PMC7109667

[CIT0013] Fonseca, V. G. L., B. I. Cappellozza, O. A. de Sousa, M. Sagawa, B. Rett, M. L. Chizzotti, and R. F. Cooke. 2021. Strategic administration of an appeasing substance to improve performance and physiological responses of Bos indicus feedlot cattle. J. Anim. Sci. 99:skab322. doi: 10.1093/jas/skab322 34734258 PMC8763227

[CIT0014] Francisco, C. L., R. F. Cooke, R. S. Marques, R. R. Mills, and D. W. Bohnert. 2012. Effects of temperament and acclimation to handling on feedlot performance of feeder cattle originated from a rangeland-based cow–calf system. J. Anim. Sci. 90:5067–5077. doi: 10.2527/jas.2012-5447 22952362

[CIT0015] Goodchild, A. V. 1985. Gut fill in cattle: effect of pasture quality on fasting losses. Anim. Sci. 40:455–463. doi: 10.1017/s0003356100040149

[CIT0016] Hervet, C., J. Boullier, M. Guiadeur, L. Michel, L. Brun-Lafleur, A. Aupiais, J. Zhu, B. Mounaix, F. Meurens, F. Renois, et al. 2021. Appeasing pheromones against bovine respiratory complex and modulation of immune transcript expressions. Animals. 11:1545. doi: 10.3390/ani11061545 34070477 PMC8229285

[CIT0017] Hilton, W. M. 2015. Management of preconditioned calves and impacts of preconditioning. Vet. Clin. North Am. Food Anim. Pract. 31:197–207. doi: 10.1016/j.cvfa.2015.03.002 26139187

[CIT0037] Holy, T. E., C. Dulac, and M. Meister. 2000. Responses of Vomeronasal Neurons to Natural Stimuli. *Science*. 289:1569–1572. doi:10.1126/science.289.5484.1569 10968796

[CIT0018] Marques, R. S., R. F. Cooke, C. L. Francisco, and D. W. Bohnert. 2012. Effects of twenty-four hour transport or twenty-four hour feed and water deprivation on physiologic and performance responses of feeder cattle. J. Anim. Sci. 90:5040–5046. doi: 10.2527/jas.2012-5425 22851237

[CIT0020] Marques, R. S., D. W. Bohnert, O. A. de Sousa, A. P. Brandão, T. F. Schumaher, K. M. Schubach, M. P. Vilela, B. Rett, and R. F. Cooke. 2019. Impact of 24-h feed, water, or feed and water deprivation on feed intake, metabolic, and inflammatory responses in beef heifers. J. Anim. Sci. 97:398–406. doi: 10.1093/jas/sky397 30312410 PMC6313126

[CIT0021] McGlone, J. J., and D. L. Anderson. 2002. Synthetic maternal pheromone stimulates feeding behavior and weight gain in weaned pigs. J. Anim. Sci. 80:3179–3183. doi: 10.2527/2002.80123179x 12542158

[CIT0022] Munck, A., P. M. Guyre, and N. J. Holbrook. 1984. Physiological functions of glucocorticoids in stress and their relation to pharmacological actions*. Endocr Rev. 5:25–44. doi: 10.1210/edrv-5-1-25 6368214

[CIT0023] National Academies of Sciences, Engineering, and Medicine, NASEM, 2016. Nutrient requirements of beef cattle model. 8th rev. ed. Washington, DC: The National Academies Press. doi: 10.17226/19014

[CIT0024] Osella, M. C., A. Cozzi, C. Spegis, G. Turille, A. Barmaz, C. L. Lecuelle, E. Teruel, C. Bienboire-Frosini, C. Chabaud, L. Bougrat, et al. 2018. The effects of a synthetic analogue of the Bovine Appeasing Pheromone on milk yield and composition in Valdostana dairy cows during the move from winter housing to confined lowland pastures. J. Dairy Res. 85:174–177. doi: 10.1017/S0022029918000262 29785915

[CIT0025] Pageat, P. 2001. Appeasing Pheromones to decrease stress, anxiety and aggressiveness. US Patent 6,169,113 B1. Jan. 2, 2001.

[CIT0026] Pageat, P., and Y. Teyssier. 1998. Usefulness of a porcine pheromone analogue in the reduction of aggressions between weanlings on penning; behavior study. In Proceedings of the 15th International Pig Veterinary Society Congress, vol. 4, p. 413. Nottingham University Press.

[CIT0034] Pescara, J. B., J. A. Pires, and R. R. Grummer. 2010. Antilipolytic and lipolytic effects of administering free or ruminally protected nicotinic acid to feed-restricted Holstein cows. *J. Dairy Sci*. 93:5385–5396. doi: 10.3168/jds.2010-3402 20965354

[CIT0028] Roeber, D. L., N. C. Speer, J. G. Gentry, J. D. Tatum, C. D. Smith, J. C. Whittier, G. F. Jones, K. E. Belk, and G. C. Smith. 2001. Feeder Cattle Health Management: Effects on Morbidity Rates, Feedlot Performance, Carcass Characteristics, and Beef Palatability1, 21This project was funded by the Kentucky Beef Council, Lexington, KY.2The authors wish to thank Excel, Inc., Division of Cargill, Fort Morgan, CO, for their assistance in conducting this project. Prof. Anim. Sci. 17:39–44. doi: 10.15232/s1080-7446(15)31566-7

[CIT0029] Schubach, K. M., R. F. Cooke, C. L. Daigle, A. P. Brandão, B. Rett, V. S. M. Ferreira, G. N. Scatolin, E. A. Colombo, K. G. Pohler, and B. I. Cappellozza. 2020. Administering an appeasing substance to beef calves at weaning to optimize productive and health responses during a 42-d preconditioning program. J. Anim. Sci. 98. doi: 10.1093/jas/skaa269 PMC8060919 32827437

[CIT0033] Van Soest, P. J., J. B. Robertson, and B. A. Lewis. 1991. Methods for dietary fiber, neutral detergent fiber, and nonstarch polysaccharides in relation to nutrition animal. *J. Dairy Sci.* 74: 3583–3597. 1660498 10.3168/jds.S0022-0302(91)78551-2

[CIT0030] Vieira, D. G., M. Vedovatto, H. J. Fernandes, E. de A. Lima, M. C. D’Oliveira, U. de A. Curcio, J. Ranches, M. F. Ferreira, O. A. de Sousa, B. I. Cappellozza, et al. 2023. Effects of an appeasing substance application at weaning on growth, stress, behavior, and response to vaccination of bos indicus calves. Animals. 13:3033. doi: 10.3390/ani13193033 37835638 PMC10571994

[CIT0035] Weary, D. M., J. Jasper, and M. J. Hötzel. 2008. Understanding weaning distress. *Appl Anim Behav Sci*. 110:24–41. doi: 10.1016/j.applanim.2007.03.025

[CIT0031] Wieringa, F. L., R. A. Curtis, and O. M. Radostits. 1974. The effect of preconditioning on weight gain and shrinkage in beef calves. Can. Vet. J. 15:309–311. 4434309 PMC1696721

